# The *MyoRobot* technology discloses a premature biomechanical decay of skeletal muscle fiber bundles derived from R349P desminopathy mice

**DOI:** 10.1038/s41598-019-46723-6

**Published:** 2019-07-24

**Authors:** Michael Haug, Charlotte Meyer, Barbara Reischl, Gerhard Prölß, Kristina Vetter, Julian Iberl, Stefanie Nübler, Sebastian Schürmann, Stefan J. Rupitsch, Michael Heckel, Thorsten Pöschel, Lilli Winter, Harald Herrmann, Christoph S. Clemen, Rolf Schröder, Oliver Friedrich

**Affiliations:** 1 Institute of Medical Biotechnology, Friedrich-Alexander-University (FAU) Erlangen-Nürnberg, Erlangen, Germany; 2 Chair of Sensor Technology, FAU Erlangen-Nürnberg, Erlangen, Germany; 3 Institute of Multi Scale Simulation of Particulate Systems, FAU Erlangen-Nürnberg, Erlangen, Germany; 4 Institute of Neuropathology, University Hospital Erlangen, Erlangen, Germany; 5 Department of Neurology, Heimer Institute for Muscle Research, University Hospital Bergmannsheil, Ruhr-University Bochum, Bochum, Germany; 6 Center for Biochemistry, Institute of Biochemistry I, Medical Faculty, University of Cologne, Cologne, Germany; 7 School of Medical Sciences, Faculty of Medicine, University of New South Wales, Wallace Wurth Building, Sydney, Australia; 8 Muscle Research Center Erlangen (MURCE), FAU Erlangen-Nürnberg, Erlangen, Germany

**Keywords:** Ageing, Biomedical engineering

## Abstract

Mutations in the *Des* gene coding for the muscle-specific intermediate filament protein desmin lead to myopathies and cardiomyopathies. We previously generated a R349P desmin knock-in mouse strain as a patient-mimicking model for the corresponding most frequent human desmin mutation R350P. Since nothing is known about the age-dependent changes in the biomechanics of affected muscles, we investigated the passive and active biomechanics of small fiber bundles from young (17–23 wks), adult (25–45 wks) and aged (>60 wks) heterozygous and homozygous R349P desmin knock-in mice in comparison to wild-type littermates. We used a novel automated biomechatronics platform, the *MyoRobot*, to perform coherent quantitative recordings of passive (resting length-tension curves, visco-elasticity) and active (caffeine-induced force transients, pCa-force, ‘slack-tests’) parameters to determine age-dependent effects of the R349P desmin mutation in slow-twitch *soleus* and fast-twitch *extensor digitorum longus* small fiber bundles. We demonstrate that active force properties are not affected by this mutation while passive steady-state elasticity is vastly altered in R349P desmin fiber bundles compatible with a pre-aged phenotype exhibiting stiffer muscle preparations. Visco-elasticity on the other hand, was not altered. Our study represents the first systematic age-related characterization of small muscle fiber bundle preparation biomechanics in conjunction with inherited desminopathy.

## Introduction

Skeletal muscle tissues are under constant active and passive mechanical stress during contraction and external stretch. The biomechanical features and stress resistance of individual striated muscle cells as well as the whole organ are complex and depend on the interconnected structural properties of sarcomeres, the extra-sarcomeric cytoskeleton, the sarcolemma, and the surrounding connective tissue^1–5^. The essential role of subcellular cytoskeletal components, in particular for the functional integrity of striated muscle cells, is underlined by the observation that mutations in genes encoding for such proteins cause various forms of myopathies and cardiomyopathies^6–8^. However, an understanding of the role of individual proteins in these cytoskeletal compartments as well as the impact of disease-causing mutations on muscle fiber and tissue biomechanics and their consequences for the disease progression is only beginning to evolve.

The present work focuses on the influence of a major component of the extra-sarcomeric cytoskeleton, i.e. the desmin intermediate filament (IF) system, on the active and passive biomechanical properties of muscle fiber bundles. The desmin three-dimensional filamentous network exerts multiple roles in the alignment and anchorage of myofibrils, the positioning of mitochondria and myonuclei, mechanosensing, stress endurance, and cell signaling^9–12^. Desmin is a bona fide IF protein with a molecular weight of 53 kDa, which in humans is encoded by the single copy *Des* gene on chromosome 2q35. Like all IF proteins, desmin exhibits a tripartite structure with a central *α*-helical coiled-coil forming domain (‘rod’) flanked by non-structured amino-terminal (‘head’) and carboxy-terminal (‘tail’) domains^10^. Desmin’s intrinsic self-assembly properties drive the multi-step formation of three-dimensional desmin filament networks. It starts with the dimerization of two desmin molecules via coil-coil formation of their *α*-helical rods; subsequently, two dimers assemble into a half-staggered antiparallel tetramer. Eight of such tetramers further associate into 60 nm-long so called “unit-length” filaments (ULF). Desmin filament growth is driven by serial longitudinal end-on annealing of individual ULFs with other ULFs and with emerging short filaments^13^. During the elongation process, long filaments will spontaneously reduce their diameter by radial compaction to form the mature desmin IF. The newly formed desmin filaments are incorporated into a complex network through attachment to various intracellular organelles and adhesion sites by a group of high molecular weight cross-bridging factors such as the various plectin isoforms and nesprins^14,15^. Notably, and in clear contrast to the actin-myosin and microtubule cytoskeletal networks, the IF system remains largely intact when exposed to large physical strains. This is because IFs have lower turnover rates and can be stretched by more than at least 350% before they rupture^16,17^. Based on these findings, it has been postulated that the IF system can act as a ‘memory’ of the cell’s organization and polarization prior to the application of large strains, thus providing a template for the re-assembly of other cytoskeletal components after a mechanical insult to the cell has ceased^18^. The deleterious effects of an abnormal desmin IF system, either due to the presence of mutant desmin or the absence of wild-type (wt) desmin protein, is highlighted by human desminopathies which comprise autosomal-dominantly and recessively inherited myopathies and cardiomyopathies^10^.

Exploiting patient-mimicking heterozygous (het) and homozygous (hom) R349P desmin knock-in mice, which harbor the ortholog of the most frequently encountered human desmin missense mutation R350P, our previous work demonstrated that the expression of mutant desmin damages striated muscle fibers and tissue already at pre-clinical disease stages by compromising the structure and function of the extra-sarcomeric cytoskeleton. As a consequence, this leads to alterations of the myofibrillar cytoarchitecture, a severe disruption of the lateral sarcomere lattice, a distortion of the myofibrillar angular axial orientation^11,12^, and an increased predisposition to stretch-induced damage in small fiber bundles.

**Figure 1:**
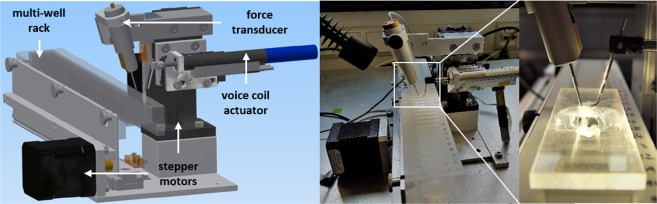
CAD design and photograph of the *MyoRobot*. Stepper motors drive the multi-well rack and lift or lower the muscle sample. The latter is mounted between force transducer and voice-coil pin (white box, enhanced to the right). The output current of the force-transducer is converted to a voltage signal and fed to a bridge amplifier prior to digitization. The voice-coil is an electro-magnetic length controller to stretch or relax the muscle fiber at *μ*m resolution.

In the present study, we employed our *MyoRobot* technology to study age-related small muscle fiber bundle biomechanics in het and hom R349P desmin knock-in mice. The *MyoRobot* (Fig. 1) is an automated biomechatronics system combining optical force transducer technology with high precision three-dimensional actuation and customized control software, thus enabling modular experimentation packages and automated data analysis in connection with RStudio^19,20^. It replaces previously used tedious manual biomechanics protocols in the context of muscle research^21^. The combination of force sensing instrumentation with precise voice-coil-driven length control allows to analyze a wealth of passive and active biomechanics properties through well defined slacks/stretches or exposure to bio-active solutions within the multi-well rack. The known finite stiffness of the transducer pin is used to calculate exerted force based on Hooke’s law as the muscle fiber contracts or relaxes while mounted between force transducer and voice-coil pin. In summary, we observed no alteration of active force properties in presence of R349P desmin, while passive steady-state elasticity (axial stiffness) was markedly increased, predominantly in fast-twitching EDL muscle fiber bundles.

## Results

### Ca^2+^-regulated active force production is not systematically altered in small fiber bundles of fast-twitch (EDL) and slow-twitch (*soleus*) muscles from R349P desmin knock-in mice

In extension to our previous study in which we assessed Ca^2+^-regulated biomechanics in small fiber bundles of SOL muscles from young (17–23 wks) mice carrying one (het) or two (hom) R349P desmin knock-in alleles^10^, we now also included young animals, adult (35–45 wks), and aged animals (~60 wks), as well as the fast-twitch EDL muscle. While general differences in the caffeine-triggered SR Ca^2+^-release force transient were found between fast- and slow-twitching fiber bundles, an age-related increase in active force appeared to have a greater influence than the presence of R349P desmin (SI Fig. 1).

**Figure 2:**
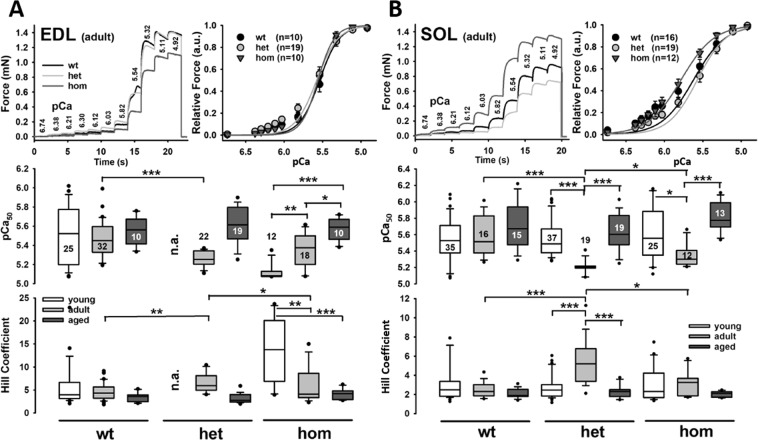
Ca^2+^ sensitivity of the contractile apparatus in small fiber bundles from (**A**) EDL and (**B**) SOL muscle of mice carrying the R349P desmin mutation during aging. Top row shows representative examples of force recordings during successive pCa steps in the adult age group (35–45 wks) for a small fiber bundle of each genotype alongside with the group analysis at this age and the mean reconstructed sigmoidal Hill fits. From Hill fits to each individual recording, the pCa_50_ values (middle row) and Hill coefficients (bottom row) were extracted and analyzed. Although not significant, there was a tendency for increased pCa_50_ values during aging across all genotypes. There were no significant differences in Ca^2+^-force sensor curve parameters by the presence of one or two R349P desmin alleles in the EDL bundles while in the SOL Ca^2+^-sensitivity was significantly lower in the mutation-carrying bundles from adult mice only. Numbers within or adjacent to the box plots indicate number of fiber bundles recorded. Box plots denominate lower and upper quartile and median value. Whiskers depict 5% and 95% percentile. Dots represent outliers. *P < 0.05; **P < 0.01; ***P < 0.001, Kruskal-Wallis with post-hoc analysis (Dunn).

Next, we assessed the Ca^2+^-biosensor properties of the contractile apparatus. Figure 2(A,B) show representative force traces during descending pCa incubations in a small bundle from EDL and SOL muscle of adult mice of each genotype (top panel, left). Also displayed are the group data for this age group showing all mean pCa-force data alongside with the mean Hill fits reconstructed from the mean pCa_50_ values and Hill coefficients obtained from each individual recording (top panel, right). For EDL bundles, the sensor curves all matched very closely, while in the SOL bundles, there was a left-shift in the hom bundles but not the het bundles, suggestive of a Ca^2+^-desensitization in adult SOL muscle. Detailed analysis from all genotypes and age groups investigated display a significant Ca^2+^-desensitization between adult wt and het SOL and EDL fiber bundles and an age-related trend (significant for hom fiber bundles) towards an increase in pCa_50_ values and decrease in Hill coefficients. This effect seems to be unrelated to the presence of mutant desmin but rather represents a fundamental effect of aging *per se*. Note that due to closing of breeding colonies at some stage, young het animals were not available to assess EDL bundles.

### Passive elasticity points towards prematurely increased biomechanical axial stiffness predominantly in fast-twitch muscle fiber bundles from R349P desmin knock-in mice

**Figure 3:**
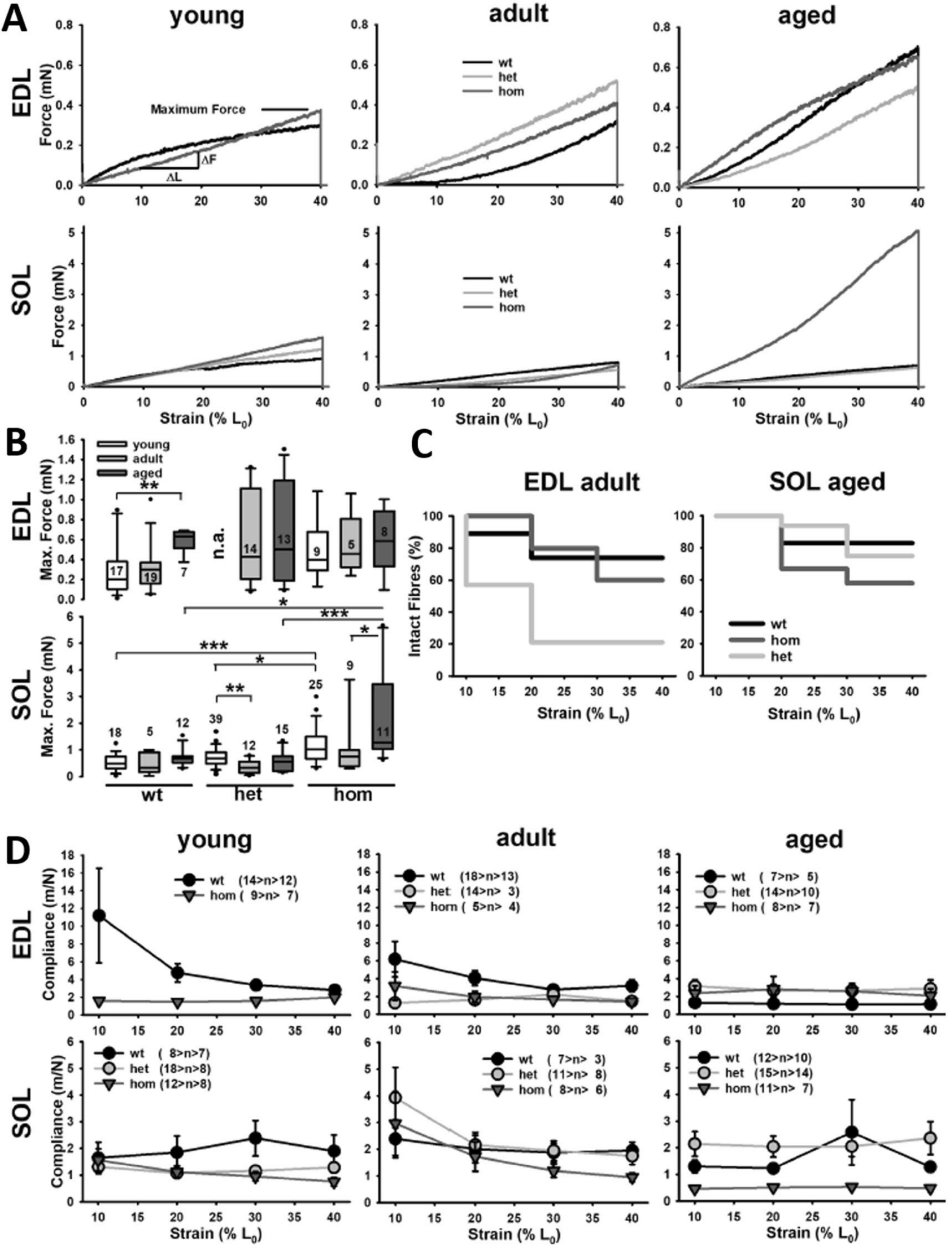
Passive elasticity and mechanical axial compliance of small fiber bundles from EDL and *soleus* (SOL) muscle of mice carrying the R349P desmin mutation during aging. (**A**) Examples of resting length-tension (RLT) curves from one fiber bundle per age group (young: 17–23 wks, adult: 35–45 wks, aged: 60–80 wks from each genotype wt, het hom). In the EDL (top) and SOL bundles (bottom), restoration forces increase with age with an emphasis on a pre-aged increase in the mutant bundles and generally larger forces in the SOL over EDL bundles (note the different scaling). (**B**) Statistical analysis shows complex significant changes in hom SOL bundles with age. (**C**) The larger restoration forces in hom and het bundles over the wt in EDL bundles are also reflected by the better survival of larger stretches in the wt. SOL bundles seem less fragile even in aged animals as compared to EDL bundles from even younger animals. (**D**) Axial absolute compliance values obtained from RLT curves for all bundles grouped by age and genotype indicate decreasing compliance with age in the EDL for wt mice, while for R349P desmin, the low compliance seen in older wt mice is already present in younger animals, indicative of a pre-aged phenotype. In SOL muscle, such a difference is not systematically seen. Numbers indicate number of fiber bundles recorded. *P < 0.05; **P < 0.01; ***P < 0.001, Kruskal-Wallis with post-hoc analysis (Dunn). Error bars = s.e.m.

To further elaborate on our previous study on reduced biomechanical axial compliance in small SOL fiber bundles in young hom R349P knock-in mice^10^, we applied the *MyoRobot* technology now to bundles of all three age-bins and including the fast-twitch EDL muscle. Figure 3(A) shows full comparative resting length-tension (RLT) curves from small fiber bundles of each genotype and age group investigated for the EDL (top) and the SOL (bottom) muscles. While for EDL bundles from young mice RLTs were very similar between genotypes, adult bundles with the R349P background became steeper and showed higher restoration forces than the wt, compatible with increased axial stiffness. In the aged cohort, wt mice then had caught up to the mutant EDL bundles. The group analysis in Fig. 3(B) confirms this increase in maximum restoration forces in wt animals with age (highly significant increase in young vs. aged EDL wt bundles). In bundles from mutant mice, maximum restoration forces already started at high levels at younger age and then only slightly increased further. The higher stiffness of EDL bundles also impacted on survival during the slow stretch protocol for which bundles with the R349P desmin background broke earlier, more so for het bundles than for hom bundles. The behavior of the maximum restoration forces is also reflected by the 10% strain-wise derived axial compliance values, summarized in Fig. 3(D), for which the biomechanical compliance was higher in young EDL bundles from wt mice over the mutant animals for all strains tested while compliance successively decreased also in the wt with age. In the mutants, compliance remained low for all ages. For SOL muscle bundles, restoration forces were slightly above wt levels in the young and adult mutants, but strikingly increased in the bundles from old hom R349P mice (A). This was also substantiated by the statistical analysis (B) and explains the overall reduced survival during stretch over het and wt SOL bundles (C). The axial compliance in SOL and EDL bundles showed tendencies for a reduced compliance in hom R349P mice over wt bundles across all ages, although not reaching statistical significance. The het bundles were in fact closer to the compliance values found in bundles from wt mice (Fig. 3D, i.e. adult and aged SOL bundles).

### Trend towards faster unloaded shortening in small fiber bundles of mutant R349P mice with age

**Figure 4:**
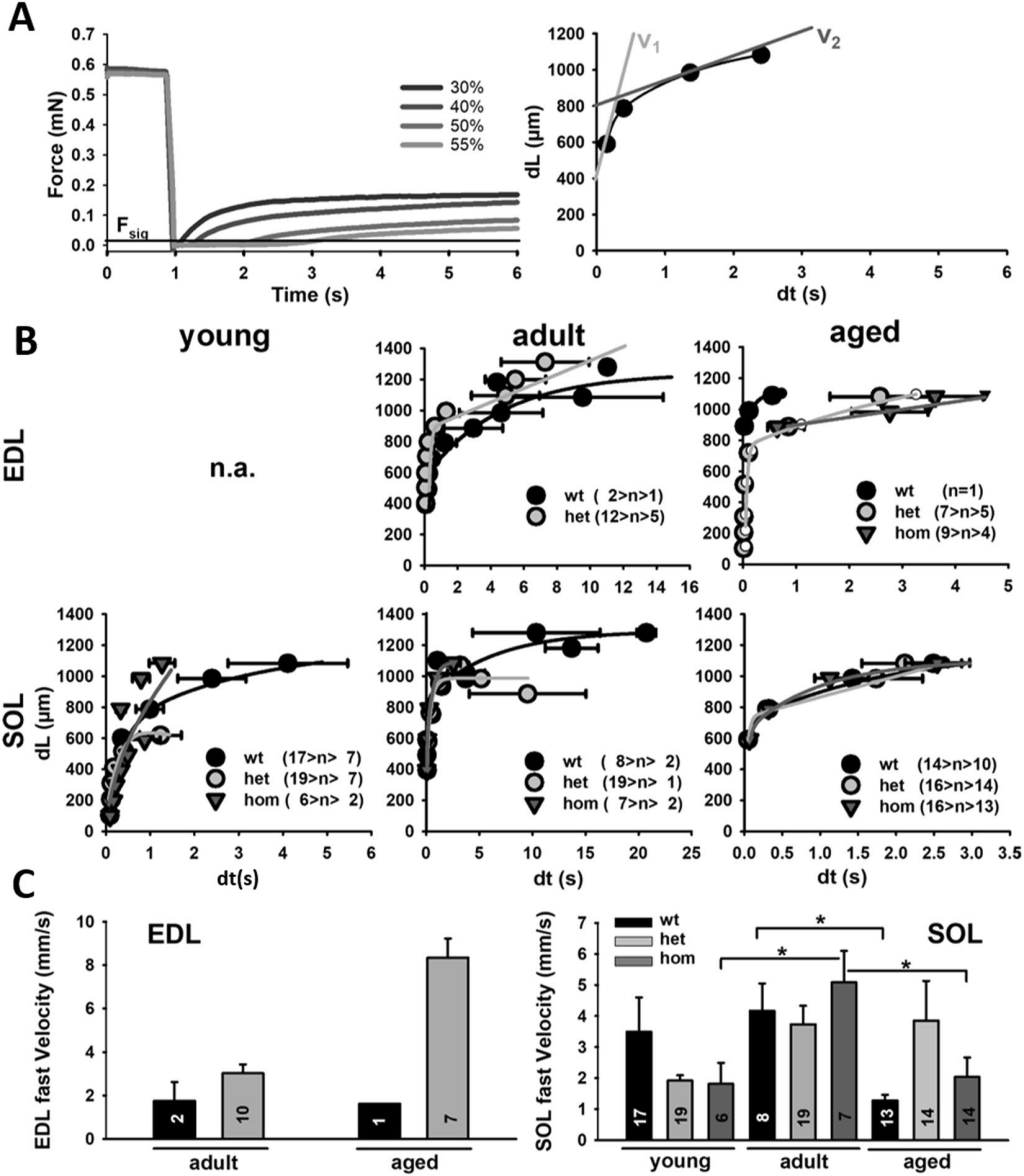
Unloaded speed of shortening in a ‘slack-test’, configuration is increased with age in small muscle fiber bundles from EDL and SOL muscle carrying the R349P desmin mutation. (**A**) Example of a slack-test performed in a small fiber bundle from SOL muscle alongside with the corresponding dL-dt plots and biexponential fits from which the linear fast (v_1_) and slow (v_2_) shortening velocities were extrapolated. (**B**) Summary of all grouped dL-dt plots across ages and genotypes. (**C**) Comparison of the fast shortening velocities representing the unloaded shortening phase in EDL and SOL bundles. Although no genotype-related statistical significances were apparent, there is a trend towards larger shortening velocities in the mutants for older ages while at young age, the wt performs faster. Numbers within or adjacent to the box plots indicate number of fiber bundles recorded. Error bars: s.e.m.

With the advantage of our *MyoRobot* technology, the rapid voice-coil actuation allowed to apply fast slacks to maximally activated fibers^19^. Thus, we aimed to perform so-called slack-tests to the available fiber bundles within the same recording sessions to extract speeds of unloaded shortening. We hypothesized that the potentially increased stiffness in mutant, i.e. hom, bundles would also be reflected by slowed-down shortening kinetics, assuming the increased axial stiffness to not only influence stretch, but also to affect taking up of a slack during an unloaded contraction. Figure 4(A) shows representative examples from one EDL and one SOL fiber bundle from an aged het R349P mouse. After reaching a plateau force during maximum Ca^2+^-activation in HA solution, slacks of increasing percentage lengths of the resting length L_0_ were suddenly applied that are answered by a drop of force to zero, redeveloping over time as the slack is being taken up again. The larger the slack dL, the longer the slack time dt, which is shown in the relations from those examples in the right panels. As described before, those relations followed a biexponential behavior, and tangential fits to the initial and the late phase allowed to extract fast unloaded (represented by v_1_) and slow internally loaded (represented by v_2_) velocities, respectively^19^. Figure 4(B) presents the dL-dt relationships obtained for all available age groups and genotypes, from which the group analyses of v_1_ and v_2_ were derived (Fig. 4(C)). Although no significant differences could be detected regarding age and genotypes (Kruskal-Wallis), there was a trend towards increased fast velocities v_1_ in the bundles with the mutation background, in particular for adult and aged but not young animals. Note that due to the implementation of slack-tests towards the end of our study, unfortunately, no more young animals and adult hom animals for EDL testing were available. Moreover, since unloaded shortening is only represented by v_1_, the slow phase v_2_ was omitted here, also because only few slacks in that dL range were performed. Taken together, unloaded speed of shortening was not significantly affected by the R349P mutation albeit it affected the stiffness in old animals to large extent.

## Discussion

We employed our *MyoRobot* technology in an age-dependent study to address the effects of the murine analogous of the human R350P desmin missense mutation^22^ in het and hom mice on the active and passive biomechanics properties in fast- and slow-twitch muscles. The most prominent effect of the R349P desmin mutation is an increased axial fiber bundle stiffness (i.e. decreased axial compliance), which was previously observed in small SOL fiber bundles from young animals^11,22,23^. The more quantitative approach using our *MyoRobot* platform yielded compliance values of ~2 m/N for SOL bundles and, in accordance to literature, larger values for fast-twitch EDL bundles^19,24^. The restoration forces recorded in young SOL fiber bundles at 140% L_0_ extension matched well with those of our exploratory study^11^ and validated our *MyoRobot* over previously tedious conventional manual biomechatronics systems^21^. The generally much larger restoration forces in SOL over EDL bundles also reflects the larger stiffness in the former. Yet, interestingly, SOL bundles still showed a better resistance to stretch-induced bundle ruptures over even younger EDL fiber bundles (Fig. 3(C)). An increased contractile stiffness was postulated to be beneficial in slow muscle where resistance to lengthening is fundamental to maintaining posture^23^. Highest values for shear moduli were also found in the SOL muscle over other lower limb deep muscles (i.e. EDL) in recent stiffness mapping experiments in humans *in vivo*^25^.

Similar to our observations in mutant desmin fiber bundles, Anderson *et al*.^26^ investigated a largely increased passive resistance to stretch in desmin knock-out mice. These findings were initially associated with alterations within the titin molecule. However, since neither titin expression nor electrophoretic mobility were altered in desmin knock-out single fibers, it was argued that the marked increase seen in passive stiffness in desmin deficient SOL muscle arose from additional extracellular elasticity components, e.g. mediated by fibrosis^27^. In accordance with a marked increase in passive elasticity in R349P desmin small fiber bundles, our previous study revealed a marked increase in extracellular collagen-I content in SOL muscles, with a hom > het > wt sequence^10^. Similar to our preliminary results in young (17–23 wks) mice bundle preparations, the visco-elastic behavior of fiber bundles does not seem to be systematically altered by the presence of mutated desmin (SI Fig. 2). This may be explained by the fact that the molecular correlate of skeletal muscle visco-elastic behavior is primarily linked to the unfolding of titin globular domains^28,29^. Also, in desmin knock-out single SOL fibers, no differences regarding viscous step extension parameters were found^27^, supporting a minor role of desmin, both for wt and mutated R349P protein, in setting visco-elastic passive muscle properties.

Active contractility showed a much lower affection by the presence of R349P desmin as compared to passive biomechanics. The tendency towards increased caffeine-induced force transient amplitudes in SOL fiber bundles from hom R349P mice of the youngest (17–23 wks) age group^11^ was also observed in the present study (SI Fig. 1(B)), but did not reach statistical significance. With the more complete picture presented here, it seems unlikely that the presence of R349P desmin negatively impacts on chemically induced SR-Ca^2+^ release-mediated force transients. If anything, there seems to be a consistent tendency for somewhat larger force amplitudes in the R349P desmin bundles, with a slightly larger force in the hom over het bundles, in both EDL and SOL muscles. Maximum Ca^2+^-saturated force was not systematically different for any condition. This suggests no obvious involvement of desmin in sarcoplasmic reticulum homeostasis, although SERCA1, RyR1 or EC-coupling function were not explicitly tested and may require future investigations. In desmin-negative adult *flexor digitorum brevis* fibers, global Ca^2+^ handling kinetics was not altered in fibers of normal (unbranched) morphology^30^ which corroborates with our conclusions that neither presence nor absence of wt or mutated desmin would actually affect sarcoplasmic Ca^2+^ handling properties, at least in unbranched fibers, which was the case in all fibers of our R349P model, both EDL and SOL^10^.

The other important factor determining active force downstream of SR Ca^2+^ handling is the Ca^2+^-sensitivity of the contractile apparatus which, was previously found to be increased in SOL fiber bundles of young animals carrying the hom R349P mutation^11^. Now with the complete picture over all age ranges and genotypes, this effect was no longer observed in the young age group. Instead, a larger scattering was seen in the hom SOL bundles. In fact, a Ca^2+^-desensitization seemed to be present in the adult R349P age group in the SOL fiber bundles which was highly significant in the presence of R349P desmin. However, this effect seemed to be again abrogated in the aged animal group. In general, myofibrillar Ca^2+^-sensitivity did not show a consistent effect in the presence of mutated desmin across all ages, but there was a tendency for an increased contractile apparatus Ca^2+^-sensitivity during aging within each genotype, both in EDL and SOL bundles. This agrees with results from 27-months-old rat single fibers showing a higher myofibrillar Ca^2+^-sensitivity in aged EDL fibers but not in SOL fibers^31^. Since all our experiments here were carried out in a standardized and automated fashion, we believe that those exhaustive results with a high level of rigor are most reliably pointing towards no specific interaction of R349P desmin with the active SR and myofibrillar-related Ca^2+^ activation of affected muscle, at neither age.

Lastly, with our *MyoRobot* system^19^, it also became possible to address the impact of R349P desmin-related increased axial passive stiffness on unloaded shortening kinetics. Similar to our previous values determined in EDL small fiber bundles from adult wt animals unrelated to this R349P desmin study^19^, the fast component of unloaded shortening measured ~8 mm/s (Fig. 4(C)) in het R349P mice. It should be noted that due to the lack of younger mice towards the end of the study, results from EDL bundles of wt littermates or hom animals were either very scarce or not available. Nevertheless, the magnitude of velocities in bundles from het animals were comparable to those in our previous study in wt animals. More complete recordings were actually available for SOL bundles which showed no systematic genotype-related significant differences for the fast shortening velocity, but rather a trend towards slower unloaded shortening in the R349P background in young animals. This was blunted for older age groups. Despite, no firm interpretation towards a change in shortening kinetics in mutated SOL bundles seems possible which suggests that the increased passive axial stiffness does not impact on the shortening kinetics of R349P muscle fiber bundles.

At present, the increased stiffness in both EDL and SOL fiber bundles of the R349P desmin mutation background cannot be unambiguously tracked down to either an effect of the mutated desmin on the cytoskeletal compliance or the associated fibrosis, or both. Hence, similar experiments on isolated single fibers are presently performed in our lab. Moreover, the engineered *MyoRobot* platform in its present form does not y*et al*low normalization of absolute force values to cross-sectional area to obtain specific forces. However, this would improve comparison with literature data. Therefore, we are currently engineering custom-designed optics to enable live image tracking of fiber (bundle) size and sarcomere length assessment in single fibers. First validation experiments already provided proof-of-concept which will be presented elsewhere and then routinely used in follow-up biomechanics studies on muscle. The aforementioned improvement has been engineered and published during the review process of the current study and can be found in Haug *et al*.^32^.

In summary, our study represents the first detailed systematic biomechanical analysis of both slow- and fast-twitch muscle fiber bundles from R349P mice during aging. The most prominent effect of the presence of mutated desmin in fully differentiated muscle preparations is a marked increase in passive axial elasticity compatible with a pre-aged phenotype in diseased muscle.

## Methods

### Animals and muscle preparations

The murine R349P desmin knock-in model B6J.129Sv-*Des*^tm1.1Ccrs^ (http://www.informatics.jax.org/allele/MGI:5708562)^22,33,34^ was used. Het animals carrying one mutated allele, hom mice carrying two mutated alleles and wt littermates were divided in age groups of young (17–23 wks), adult (35–45 wks) and aged (60–80 wks) animals, as in our previous morphometry study^11^. EDL (a typical fast-twitch muscle) or SOL muscle (a slow-twitch muscle) were isolated from the hind limbs of male and female mice after sacrifice of the animals. All animal handling and sacrifice procedures were according to approved animal experimentation guidelines at the University of Erlangen-Nürnberg (TS-6/2016) and followed the guidelines of the Federation of European Laboratory Animal Science Associations. EDL and SOL isolation was performed under a binocular, utilizing fine forceps and scissors, before the separated muscles were pinned onto a Polydimethylsiloxane (PDMS) coated Petri dish under slight stretch and newly submerged in cold Ringer’s solution. After incubation for 30 minutes, Ringer’s solution was exchanged for Ca^2+^-free, high K^+^ solution (HKS) to permanently depolarize membrane potential and inactivate Na^+^ channels after an initial isometric contraction. Fascicles were separated and small fiber bundles (~5 fibers) tethered and then transferred to the *MyoRobot* in an HKS droplet on a glass slide, positioned on top of the multi-well rack below the pins of FT and VC and manually lifted out of the droplet, loosely wrapped around the two pins and then quickly glued at both ends using a cellulose-acetate-based glue before lowering them in the *idle* well underneath, containing low relaxing solution (LR) with 0.5% HR.

### Chemical solutions

*Ringer*'*s solution* contained (in mM): 145 NaCl, 5 KCl, 2.5 CaCl_2_, 10 Hepes (4-(2hydroxyethyl)-1-piperazineethanesulfonic acid), 1 MgCl_2_, 10 glucose, pH 7.4 and osmolality 330 mosmol/kg. *High K*^+^ *solution*: 140 K-glutamate, 1 MgCl_2_, 10 Hepes, 1 EGTA (ethylene glycol-bis(*β*-aminoethyl ether))-N,N,N’,N’-tetraacetic acid), 10 glucose, pH 7.0, osmolality 275mosmol/kg. To chemically activate the fiber bundles, various Ca^2+^-buffered internal solutions were used. *Low relaxing* (LR) solution is a Ca^2+^-free buffering solution containing the low affinity Ca^2+^ buffer HDTA and was used after exposure to high-affinity Ca^2+^-chelating EGTA containing solutions to remove EGTA before performing SR calcium releases. LR constains: 87.7 K-glutamate, 7.86 Mg(OH)_2_, 30 Hepes, 0.4 EGTA, 6.6 HDTA (hexamethylenediaminetetraacetic acid), 8 Na_2_ATP, 10 Na_2_CP (creatine phosphate), pH 7.0, osmolality 290 mosmol/kg. *Ca*^2+^ *release solution* (RS) is LR supplemented with 30 mM caffeine, which at these concentrations, completely opens the SR Ca^2+^ release channels (ryanodine receptor type 1, RyR1)^35^. *Saponin* solution, to chemically skin muscle fibers, was made by adding saponin (0.1% w/v) to LR. *High activating* (HA) solution is a highly EGTA-buffered Ca^2+^-saturated solution that maximally activates troponin C and causes immediate force production. It contains 30 Hepes, 6.05 Mg(OH)_2_, 30 EGTA, 29 CaCO_3_, 8 Na_2_ATP, 10 Na_2_CP, pH 7.0 adjusted with 1 M KOH, osmolality 165 mosmol/kg. The concentration of free divalent cations in the buffered solution was calculated using the program React (Godfrey Smith, University of Glasgow). Free Ca^2+^ ([Ca^2+^]_free_) and Mg^2+^([Mg^2+^]_free_) in HA computed to ~12 *μ*M and 0.3 mM, respectively. *High relaxing* (HR) solution represents a highly EGTA-buffered Ca^2+^-free solution that is applied every time the preparation was exposed to Ca^2+^ ions to ensure buffering. HR contains 30 Hepes, 6.25 Mg(OH)_2_, 30 EGTA, 8 Na_2_ATP, 10 Na_2_CP, pH adjusted to 7.0 using 1 M KOH, 161 mosmol/kg. For HR, [Ca^2+^]_free_ = 1 nM and [Mg^2+^]_free_ = 0.3 mM. Likewise, React was used to calculate [Ca^2+^]_free_ for any mixtures of HA:HR used in the calcium sensitivity assessment protocol, reaching from pCa = 9 (HR) to pCa = 4.92 (HA). *Loading solution* (LS) consisted of a 3 + 7 of HA + HR to provide [Ca^2+^]_free_ ~15 nM ([Ca^2+^]_total_ ~7.7 mM) to (re)load the SR at defined immersion times. All internal solutions were freshly prepared adding creatine kinase (Sigma/Roche, Germany) dissolved in 20 mM Hepes, pH 7.0, ~300 U/ml, at the day of experiments.

### Automated *MyoRobot* force transducer biomechatronics experiments

For automated and highly objective execution of the plethora of recordings assessing the biomechanical active and passive properties of the muscle fiber bundle preparations in a high number of samples, we used our recently engineered *MyoRobot* system^19^. The biomechatronics platform is equipped with precise voice-coil actuators and sensitive force transducer sensor technology. The force sensor is operated on an optical sensor principle (FT, TR5S, Myotronic, Heidelberg, Germany), while axial movements to the fiber bundle preparation (stretch, slacks) are executed via a linear VC actuator element (VCA, CAL 12–010–51, SMAC Inc., Maccon GmbH, Munich, Germany) that allowed a 1 *μ*m position resolution and featured a LabView-implementable controller (LAC-10, Maccon). The FT-VCA array is mounted on a stage that is actuated up and down with a stepper motor (QSH4218-35-26, -40-033, Trinamic Motion Control, Hamburg, Germany) while exchanging the bath underneath the sensor-actuator block is automatically executed via another stepper motor carrying a multi-well bath sledge. Details on the mechanical engineering of the system are given in^19^. FT data were digitized using an A/D-D/A converter (NI USB-6008, National Instruments, Munich, Germany) USB-connected to a Windows laptop running LabView (National Instruments). Sensor data were continuously recorded and displayed for visual control in the GUI featuring buttons and controls to either setup a recording sequence, or manually operate the *MyoRobot* for fiber bundle mounting etc. using a self-written LabView program environment. Recording protocols could be created or loaded in a tabulator display control containing the well number # and well time t# that was translated into actuator commands, while a separate window with advanced options was used to setup VC movement. These allowed implementing a delay time, altering pin displacement in % of L_0_, to define the time it maintained its new position or to change its velocity by modifying a length difference over time. Based on a well-number (#) - exposure time (t#) matrix, the *MyoRobot* was set up to assess active and passive biomechanics properties in a sequence of different protocols. Initially, all small fiber bundles were permeabilized by immersion in saponin solution for 60–90 s and consequently washed by repeated dipping in LR. Prior to initializing the recording protocols, the SR was completely emptied from endogenous releasable Ca^2+^ in RS for 60 s, excess Ca^2+^ buffered in HR for 60 s and the preparation returned to the *idle* well.

The various biomechatronics protocols were as follows:

1. *Caffeine-induced force transients and maximum force assessment*. Intended as an initial functionality test, the emptied SR was loaded under defined conditions and subsequently, Ca^2+^ was released by caffeine exposure, followed by a maximum chemical activation according to the following sequence: 90 s LS - 1 s HR - 60 s LR - 60 s RS - 10 s HA - 60 s HR. SR loading time was chosen to be 90 s for both muscles, despite the EDL is known to operate at ~60% maximum Ca^2+^ physiological filling capacity^34^, to emphasize its improved force production under similar circumstances. SI Fig. 1A illustrates the procedure.
2. *Ca*^2+^ *sensitivity assessment of the contractile apparatus*. To assess the Ca^2+^ biosensor at the chemico-mechanical interface of troponin C, small fiber bundles were directly and successively exposed to wells containing highly-EGTA buffered solutions with decreasing pCa value. Each step lasted for 20 s allowing force to plateau before proceeding directly to the subsequent well of which at least eight different pCa values were covered for proper analysis (example traces shown in Fig. 2).
3. *Resting length-tension (RLT) curves, passive axial compliance*. Assessing the fiber bundle’s axial compliance, the VCA had to be operated at optimized velocities, slow enough to be in balance between instantaneous elastic restoration force and viscous relaxation. Furthermore, fiber bundles were kept under relaxing (LR) conditions throughout the protocol, while passive restoration force was sampled at 200 Hz. Typical RLT-recordings are displayed in Fig. 3(A).
4. *(Unloaded) speed of shortening, slack test*. The slack test reveals a two-phased velocity response when imposing sudden and increasing slacks upon a fiber isometrically activated to its maximum^19^. While being held at a resting length L_0_, the bundle was transferred to HA solution, which caused a rise in force to a steady-state. Then, the VCA was driven at maximum velocity to move in the direction of the FT pin for a given slack length (20–55% L_0_), as force quickly dropped to zero. Maintaining this new resting length allowed the fiber to reestablish isometric force, which was recorded at 2 kHz sampling rate until a second force plateau was reached. Subsequently, the preparation was moved to HR, allowing restoration of L_0_ under relaxing conditions and repeating the procedure for the next slack length.
5. *Visco-elasticity*. To characterize visco-elastic elements within the bundles, sudden stretch length changes are required. The preparation was kept in relaxing HR solution, while bundle length was sequentially increased in pre-defined steps of 10% up to 60% L_0_. Each new elongation was held for 7 s to elucidate the bundle’s viscous force relaxation, before another change of L_0_ was imposed.

### Data analysis and statistics

Using a common data format, *MyoRobot* control software enables communication with analysis routines written in RStudio (RStudio Inc., rstudio.com) and plotting/statistics tools provided by SigmaPlot (Systat Software Inc., sigmaplot.co.uk). Common for all analyses was that data files containing the values for each respective recording step were sequentially read-out and smoothed by applying a moving average filter with a window size of a tenth of the sampling frequency. For *pCa-force* recordings, the plateau force of each data file (representing a single pCa value, each) close to the end was determined, stored in a matrix alongside the pCa value and fitted to a four parameter Hill-equation using least-square method. The equation states: $y={y}_{0}+\frac{a\ast {10}^{-bx}}{{c}^{b}+{10}^{-bx}}$, while constraining *a* = 1 and *y*_0_ = 0 as boundary conditions. From the fit, steepness (b, Hill-coefficient) and pCa_50_ (−*log*_10_(*c*), inflection point) were derived for every recording to reconstruct a mean fit to the averaged data points (Fig. 2). The *axial compliance* was computed in segments of 10% L_0_, until fiber bundle rupture occurred or 140% L_0_ was reached, by fitting a slope to each section whose steepness equals the axial elasticity (the inverse of axial compliance). Analysis of the *slack test* consisted of determining the maximum regenerated isometric force of the first slack (~20% L_0_) and computing a 5% cut-off criterion as an indicator for significant force regeneration for this and all successive slacks. The time the bundle required to overcome this threshold is called slack time dt and stored in a matrix together with the respective slack length, while a bi-exponential equation, following $y=a\ast (1-{e}^{{\kappa }_{1}\ast dt})+c\ast (1-{e}^{{\kappa }_{2}\ast dt})$, was fitted to the data points. Derivation of this equation allows obtaining its steepness and thus, velocity values for the fast, unloaded phase (typically below 45% L_0_), and the slow, internally dampened phase (above 45% L_0_) based on the average slack length of each regime. To evaluate the *visco-elastic* properties, absolute restoration force and force relaxation were derived. Therefore, the baseline (*F*_0_) was determined to be the last 5 s before the first stretch and the force trace was cut in segments, corresponding to the stretches of 10% L_0_, to compute their maximum and minimum force, which was required for absolute restoration force (${F}_{abs}=ma{x}_{n\ast 10 \% }-{F}_{0}$) and force relaxation (${F}_{relax}=ma{x}_{n\ast 10 \% }-mi{n}_{n\ast 10 \% }$). Statistics were done by applying Kruskal-Wallis tests (testing age and genotype as variables) following post-hoc analysis (Dunn) in SigmaPlot.

## Supplementary information

Supplementary Info
