# Supplementary material for: The MyoRobot technology discloses a premature biomechanical decay of skeletal muscle fiber bundles derived from R349P desminopathy mice
Source: Sci Rep. 2019 Jul 24;9:10769. doi: 10.1038/s41598-019-46723-6 (PMC6656739; doi:10.1038/s41598-019-46723-6)
Supplement: Supplementary file 1 — Supplementary Info [file 41598_2019_46723_MOESM1_ESM.pdf]

The *MyoRobot* technology discloses a premature biomechanical decay of skeletal muscle fiber bundles derived from R349P desminopathy mice

Michael Haug[1,*], Charlotte Meyer[1,*], Barbara Reischl[1,*], Gerhard Prölß[1], Kristina Vetter[1], Julian Iberl[1], Stefanie Nübler[1], Sebastian Schürmann[1], Stefan J Rupitsch[2], Michael Heckel[3], Thorsten Pöschel[3], Lilli Winter[4], Harald Herrmann[4], Christoph S Clemen[5,6], Rolf Schröder[4,8], and Oliver Friedrich[1,7,8,+]

[1]Institute of Medical Biotechnology, Friedrich-Alexander-University (FAU) Erlangen-Nürnberg, Paul-Gordan Str. 3, 91052 Erlangen, Germany
[2]Chair of Sensor Technology, FAU Erlangen-Nürnberg, Germany
[3]Institute of Multi Scale Simulation of Particulate Systems, FAU Erlangen-Nürnberg, Germany
[4]Institute of Neuropathology, University Hospital Erlangen, Schwabachanlage 6, 91054 Erlangen, Germany
[5]Department of Neurology , Heimer Institute for Muscle Research, University Hospital Bergmannsheil, Ruhr-University Bochum, Bochum, Germany
[6]Center for Biochemistry, Institute of Biochemistry I, Medical Faculty, University of Cologne, Cologne, Germany
[7]School of Medical Sciences, Faculty of Medicine, University of New South Wales, Wallace Wurth Building, Sydney, NSW 2052, Australia
[8]Muscle Research Center Erlangen (MURCE), FAU Erlangen-Nürnberg, Germany
[*]these authors contributed equally to this work
[+]corresponding author: oliver.friedrich@fau.de

Supporting Information

Ca^{2+}-regulated active force production is not systematically altered in small fiber bundles of fast-twitch (EDL) and slow-twitch (*soleus*) muscles from R349P desmin knock-in mice

SI Fig. 1 shows a comparison of caffeine-induced sarcoplasmic reticulum (SR) Ca^{2+}-release-mediated force transients and maximum Ca^{2+}-saturated force in small fiber bundles of both SOL and EDL muscle from young (17 - 23 wks), adult (35 - 45 wks) and aged (∼ 60 wks) het and hom R349P desmin knock-in mice and wt littermates. SI Fig. 1(A) shows representative force transients during 30 mM caffeine incubation (SR Ca^{2+}-release) revealing a transient increase and decline in force as Ca^{2+}-ions are released, bind to the contractile apparatus, unbind after a period and are then pumped back into the SR, followed by a maximum force activation by saturating the contractile apparatus with Ca^{2+} (pCa 4.92) approx. 60 s later. As expected from slow- vs. fast-twitch muscles, the transient kinetics is somewhat slower in the SOL than in the EDL. Notably, the peak amplitudes during the caffeine-transient (SI Fig. 1B), the maximum force levels (SI Fig. 1C) and the ratio of both amplitudes (SI Fig. 1D), which is a measure for SR filling with releasable Ca^{2+} ions[1], showed no systematic differences in any variable regarding age or genotype in EDL bundles. In the analysis of SOL bundles, no systematic differences were noted with the exception of a significantly decreased maximum force in adult hom versus age-matched wt control mice, and a significant increase in maximum force from adult to aged hom animals.

Passive elasticity points towards prematurely increased biomechanical axial stiffness predominantly in fast-twitch muscle fiber bundles from R349P desmin knock-in mice

Apart from assessing steady-state elasticity, visco-elastic behavior was also assessed (see SI Fig. 2). Unlike axial biomechanical compliance, axial viscosity, as reflected by force relaxation amplitudes in response to an applied sudden stretch, was not significantly affected, except for showing a trend towards higher viscous flow in the mutants in the SOL bundles (SI Fig. 2(C)).

References

1. Lamb, G. D., Cellini, M. A. & Stephenson, D. G. Different ca2+ releasing action of caffeine and depolarisation in skeletal muscle fibres of the rat. *J. Physiol.* **81**, 715–728 (2001).

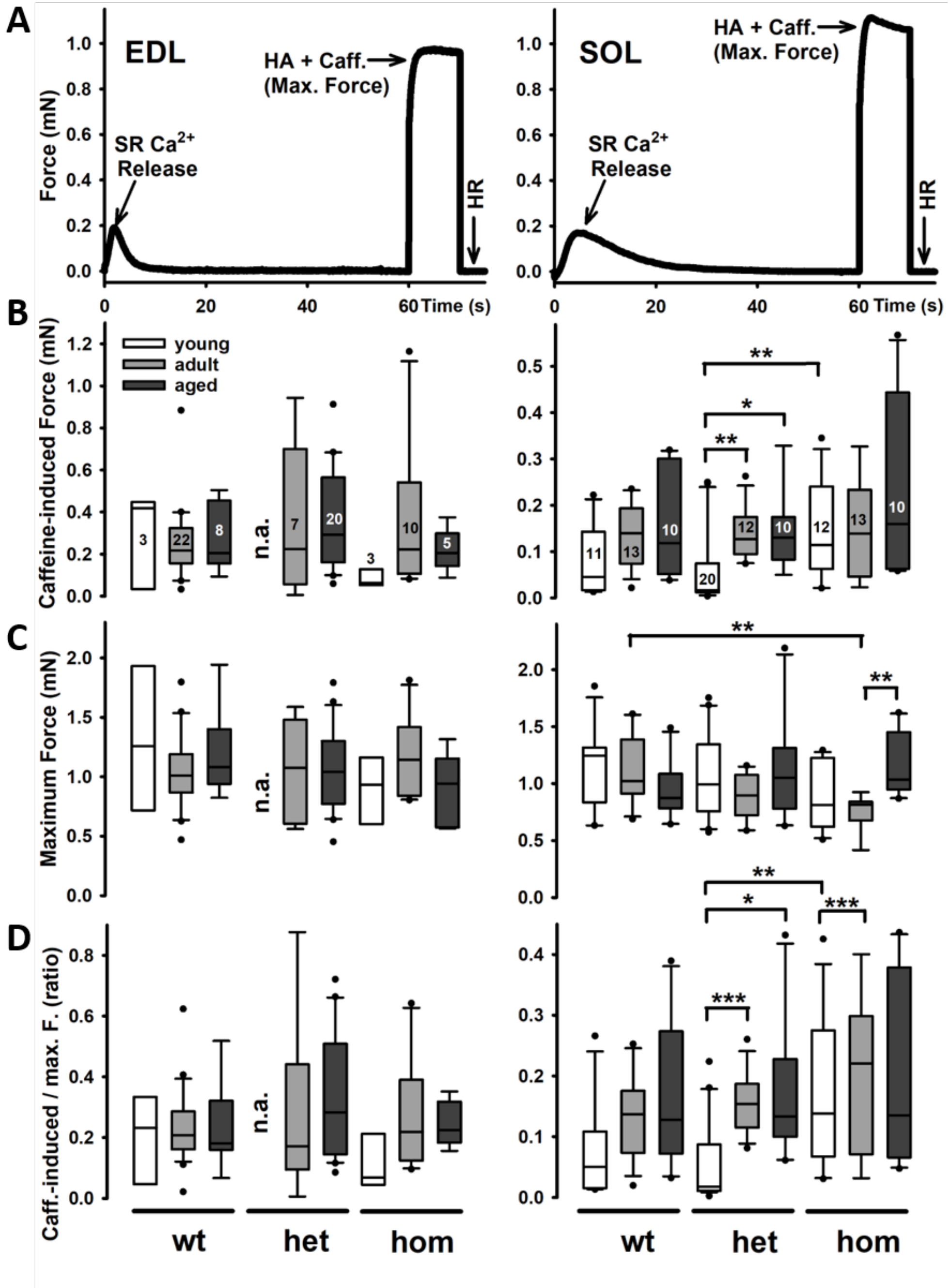

SI Fig. 1. Caffeine-induced SR Ca^{2+}-release mediated force transients and maximum Ca^{2+}-saturated force are not systematically affected by the R349P desmin mutation during aging in small fiber bundles from both EDL and SOL muscles. (**A**), representative examples of force transients evoked by 30 mM caffeine triggered release of SR Ca^{2+} pools in a small EDL or SOL fiber bundle followed by maximum Ca^{2+}-saturation of the contractile apparatus (HA+Caff.). Group analysis of (**B**) peak amplitude during caffeine SR Ca^{2+} release, (**C**) maximum force during the HA+Caff and (**D**) the ratio of both. Numbers within or adjacent to the box plots indicate number of fiber bundles recorded. Box plots denominate lower and upper quartile and median value. Whiskers depict 5 % and 95 % percentile. Dots represent outliers. *: $P < 0.05$ Kruskal-Wallis with post-hoc analysis (Dunn). There was no systematic effect of neither age nor genotype on the SR Ca^{2+} release-induced or Ca^{2+}-saturated maximum force amplitudes. n.a.: not available.

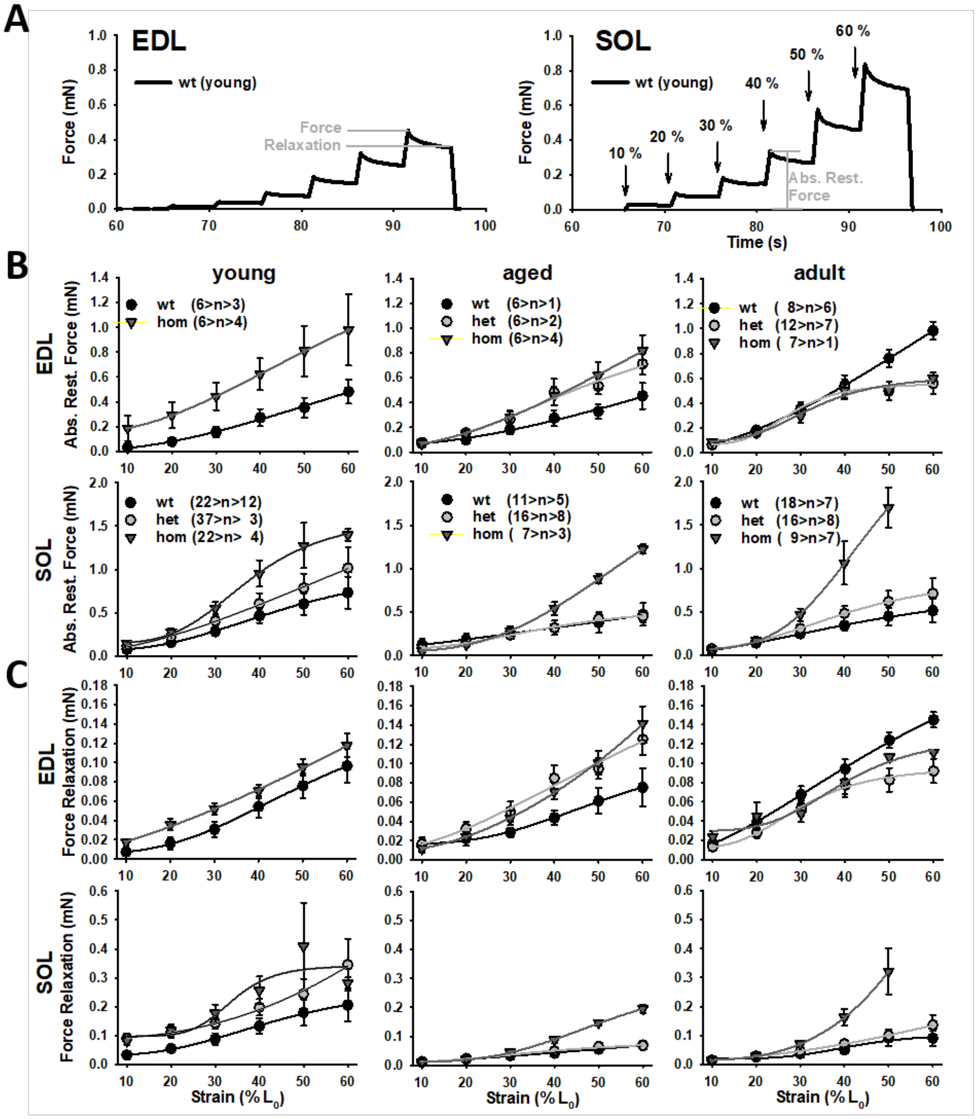

SI Fig. 2. Visco-elastic behavior of small muscle fiber bundles from EDL and SOL muscle carrying the R349P desmin mutation during aging of mice. (**A**), representative examples of quick step-stretch experimental protocols suddenly stretching the bundles in 10 % L_0 bin and recording the passive force. (**B**), group analysis across ages in EDL (top) and SOL (bottom) bundles showing increased absolute restoration force levels in the mutants over the wt for almost all ages, in accordance with the resting length-tension data (Fig. 3). (**C**), group analysis of viscous relaxation indicates similar viscosity levels among genotypes for all ages with a trend towards higher viscous flow in the mutants, in particular in the SOL bundles. Error bars: s.e.m.
